# Preventive effects of the aqueous extract of *Cichorium intybus* L. flower on ethylene glycol-induced renal calculi in rats

**Published:** 2018

**Authors:** Mahdieh Zaman Emamiyan, Gholamhassan Vaezi, Maryam Tehranipour, Khdije Shahrohkabadi, Abdolhossein Shiravi

**Affiliations:** 1 *Department of Biology, Damghan Branch, Islamic Azad University, Damghan, Iran*; 2 *Department of Biology, Faculty of Sciences, Sciences and Research Branch, Islamic Azad University, Tehran, Iran*; 3 *Department of Biology, Mashhad Branch, Islamic Azad University, Mashhad, Iran*; 4 *Molecular Genetic PhD, Department of Biology, Mashhad Branch, Islamic Azad University, Mashhad, Iran*

**Keywords:** Cichorium intybus L., Flower, Renal calculi, Ethylene glycol

## Abstract

**Objective:**

Urolithiasis remains a global problem. Despite the availability of numerous methods, no definite therapeutic agent has been yet introduced for the prevention or treatment of kidney stones. In this study, we evaluated the possible preventive effects of aqueous extract of *Cichorium intybus* L. (chicory) flowers on ethylene glycol-induced renal calculi in rats.

**Materials and Methods:**

A total of 24 Wistar rats were randomly divided into four groups and were treated for 30 days. Group A received drinking tap water, while groups B, C, and D were administered with 1% ethylene glycol for induction of calcium oxalate stone formation. Rats in groups C and D received intraperitoneal injections of the aqueous extract of chicory flowers (50 and 200 mg/kg, respectively) since the first day of the experiment. The urine volume, urine pH, and urinary levels of oxalate, citrate, calcium, uric acid, and creatinine as well as serum levels of calcium, uric acid, and creatinine were measured. After 30 days, the rats' kidneys were removed and prepared for histological evaluation of calcium oxalate deposits. One-way analysis of variance (ANOVA), followed by Tukey's test, was performed, using SPSS version 20.

**Results:**

The number of calcium oxalate crystals was significantly higher in group B (ethylene glycol-only treated animals), compared to group A (control), group C (50 mg/kg of aqueous extract), and group D (200 mg/kg of aqueous extract) (p<0.05). On day 30, the urine level of citrate, oxalate (p>0.05), and creatinine (p<0.05), as well as urine pH (p<0.05) decreased in groups C and D, compared to group B. Also, urine calcium level, urine uric acid (p>0.05), and urine volume (p<0.05) were higher in group D, compared to group B. In addition, the serum level of calcium, creatinine (p<0.05), and uric acid (p<0.001) decreased in groups C and D.

**Conclusion:**

The aqueous extract of chicory flower (50 mg/kg) could reduce the number of calcium oxalate deposits in the urine and reduce the level of serum parameters.

## Introduction

Ethylene glycol (EG) can induce inflammation, renal damage, and calcium oxalate (CaOx) kidney stones. CaOx causes oxidative damage through production of reactive oxygen species, such as superoxide and hydrogen peroxide (H_2_O_2_) (Aslan and Aksoy, 2015 ▶). Pro-inflammatory cytokines are released at the onset of inflammation to increase the release of other cytokines and activate inflammatory cells. The level of interleukin-18 (IL-18), as a mediator of acute renal damage, increases in the proximal tubular epithelium in case of renal damage and acute renal failure. Administration of EG increases tumor necrosis factor-alpha (TNF-α) and IL-18 levels in the plasma and kidneys (Aslan and Aksoy, 2015 ▶).

Urinary calculi are the third most prevalent disorder of the urinary tract, leading to urinary tract obstruction, hydronephrosis, urinary infections, and hemorrhage (Khajavi Rad et al., 2011 ▶). Extracorporeal shock wave lithotripsy, percutaneous nephrolithotomy, transurethral lithotripsy, and even laparoscopy are widely used for removal of renal calculi. However, these invasive procedures are not cost-effective and may lead to severe complications (Khajavi Rad et al., 2011 ▶). 

Various inorganic and organic promoters of CaOx crystal aggregation, such as oxalate, uric acid, and calcium, have been introduced, while inhibitors include urinary macromolecules, e.g. Tamm-Horsfall protein (THP), urinary prothrombin fragment 1 (UPF1), citrate, and magnesium (Sridharan et al., 2015 ▶). Crystal adhesion which leads to epithelial damage is an important stage of urolithiasis (i.e. formation of stones in the urinary tract). Also, oxidative stress caused by CaOx crystals and high oxalate load can lead to interstitial inflammation (Sridharan et al., 2015 ▶).

Diverse biological properties of herbal extracts have encouraged medical experts and researchers to use medicinal plants as safe treatment options for kidney stone disease. In general, significant therapeutic effects of plants are attributed to their antioxidant compounds (Sridharan et al., 2015 ▶). Therefore, it seems logical to replace conventional treatments with medicinal plants (Khajavi Radet al., 2011 ▶).

*Cichorium intybus* L. is a plant of different types with a bright blue, white, or pink color (Zaman and Nooul Basar, 2013 ▶). Wild chicory (*Cichorium intybus* L.) is a blue-flowered composite plant (De Kraker et al., 2001 ▶). Chicory flower is used as a tonic and appetite stimulant and can be applied as a herbal treatment of common ailments and gallstones (Street, et al., 2013 ▶; Katiyar et al., 2015 ▶). Moreover, it is used as a nephroprotective agent (Zaman and Noorul Basar, 2013 ▶). 

Chicory flower contains ﬂavonoids, essential oils, and anthocyanins, which contribute to the blue color of its perianth (Street et al., 2013 ▶; Katiyar et al., 2015 ▶; Eslami, 2015 ▶; Mehmood et al., 2012 ▶). In addition, this plant contains crystalline glucosides (Shaikh et al., 2010 ▶), inulins, vitamins, and minerals, which make it a mild and bitter tonic agent (Nandagopal and Ranjitha Kumari, 2007 ▶; Katiyar et al., 2015 ▶). 

The presence of CaOx crystals in the urine and development of renal failure are the characteristics of EG poisoning (Pomara et al., 2008 ▶). Also, low 24-hour urine volume, low urine pH, and increased levels of urine calcium, oxalate, and urate are known to promote stone formation (Gupta et al., 2011 ▶). Having this background in mind, in the present study, we investigated the effects of aqueous extract of chicory flower on the prevention of kidney stones in tissues of Wistar rats.

## Materials and Methods

Chicory flowers were collected from Asadabad Village, Neyshabur, Iran. The samples were identified by botanical experts at the Research Institute of Plant Sciences, Ferdowsi University of Mashhad, Mashhad, Iran, and kept in the herbarium: *Cichorium intybus* L.; family: Asteraceae (also called Compositae); with the voucher No. 38201 (FUMH).

**Extraction method**

Chicory flowers were dried in shade and maintained in a cool environment until extraction; the flowers were completely ground in an electronic mill. Afterwards, 100 g of the ground powder was dissolved in 1000 mL of water and placed in an oven for 72 hr at 35°C. The resulting mixture was filtered, using a Buchner funnel and a vacuum pump and dried in an oven at 35°C. The dried powder was used to prepare 50 and 200 mg/kg/BW concentrations. It should be noted that the extracts were prepared each day and animals received freshly prepared doses (Fatemi Tabatabaei et al., 2016 ▶; Khksari et al., 2000 ▶).

A total of 24 male Wistar rats, with the mean weight of 250±10 g, were randomly divided into four groups of six rats (of the same age) as follow: group A (control group), group B (received 1% EG) (Hadjzadeh et al., 2007 ▶ ;Shafaeifar et al., 2011 ▶), group C (low-dose preventive group that received 50 mg/kg/BW of the aqueous extract), and group D (high-dose preventive group that received 200 mg/kg/BW of the aqueous extract) (Jamshidzadeh et al., 2006 ▶). The rats were maintained in an animal room at 23±2°C with 12 hr: 12 hr light:dark cycles. The animals had *ad libitum* access to water and food and were treated for 30 days (Mehrabi et al., 2016 ▶).

**Treatment**

Over a period of 30 days, 1% EG was added to the drinking water of all groups, with the exception of group A. Also, groups A and B were administered with distilled water intraperitoneally. Groups C and D received intraperitoneal injections of aqueous extract of chicory (50 and 200 mg/kg/BW, respectively) for 30 days (Khajavi Radet al., 2011 ▶; Hadjzadeh et al., 2007 ▶). 

**Collection of urinary and blood samples and biochemical analysis**

For the evaluation of urinary parameters determining kidney stone formation, the rats were individually placed in a metabolic cage on days 0, 15, and 30 of the experiment. After 24 hr, volume of the collected urine was measured, and the samples were transferred to the laboratory for assessment of urine oxalate, citrate, calcium, uric acid, creatinine, urine volume, and urine pH. Additionally, on day 31, following ether-induced anesthesia, blood samples were directly drawn from the heart and sent to the laboratory for the measurement of serum levels of calcium, uric acid, and creatinine.

**Pathological analysis**

The kidneys were placed in formalin 10% for tissue fixation (Khajavi Radet al., 2011 ▶; Hadjzadeh et al., 2007 ▶). Microscopic slides (5-µm thick) were obtained from different regions of the rats' kidneys and stained using hematoxylin and eosin (H&E); the slices were studied under a light microscope. In each slide, 10 microscopic fields (×40 magnification) were randomly selected, and CaOx crystal deposits were counted (Khajavi Radet al., 2011 ▶; Hadjzadeh et al., 2007 ▶).

**Statistical analysis**

The data are presented as mean±standard error. After determining the normal distribution of the data, one-way analysis of variance (ANOVA), followed by Tukey's test, was performed using SPSS version 20. A p-value less than 0.05 was considered statistically significant.

## Results

**Biochemical analysis of urine samples**

Based on the results, 24-hr urine CaOx, uric acid, creatinine, citrate, oxalate and pH were almost identical among the groups, and no significant differences were observed on day 0 (p>0.05; Tables. 1, 2 and 3). Urinary CaOx increased and decreased in groups D and C, in comparison with group B on days 30 and 15, respectively. These changes were significant on day 15 (p<0.01; Table 1). On the other hand, on days 15 and 30, no significant difference was observed in uric acid (p>0.05; Table 1).

Urine creatinine level decreased in groups C and D versus groups A and B; the difference was significant in group D on day 30 (p<0.05; Table 2). On the other hand, urinary citrate level increased in group B on days 15 and 30 in comparison with other groups; the change was significant on day 15 (p<0.001; Table 3). Moreover, on days 15 and 30, urinary oxalate level in groups C and D was different from that of group B; the difference was significant on day 15 (p<0.01; Table 2).

On day 30, there was a significant increase in the urine volume in groups C and D versus group B (p<0.05; Table. 3). On day 30, urine pH reduced in all groups in comparison with group B, the difference was significant in group A in comparison with group B (p<0.05; Table 3). However, on day 15, the decline in urine pH in group D was not significantly different from other groups (p> 0.05; Table 3).

**Table 1:** Changes in 24-hr urine calcium and uric acid concentrations (mg/dl) in rats ( data expressed as mean±standard error).

	**Multiple Comparisons (Turkey Test)**				**ANOVA**
**Urinary parameters **	**Group A**	**Group B**	**Group C**	**Group D**	**P-value**
**Calcium, day 0 (mg/ dl)**	1.16±0.12	0.85±0.11	1.29±0.17	1.56±0.44	p>0.05
**Calcium, day 15 (mg/ dl)**	0.37±0.03	0.91±0.16	0.64±0.16	*1.22±0.2	p<0.01
**Calcium, day 30 (mg/ dl)**	0.57±0.28	1.24±0.18	0.46±0.1	1.51±0.54	p>0.05
**Uric acid, day 0 (mg/ dl)**	1.23±0.15	1.31±0.09	1.36±0.38	0.65±0.11	p>0.05
**Uric acid, day 15 (mg/ dl)**	0.41±0.15	0.57±0.13	0.38±0.1	0.62±0.03	p>0.05
**Uric acid, day 30 (mg/ dl)**	0.23±0.13	0.66±0.2	0.11±0.05	0.68±0.32	p>0.05

The significance level in groups C and D compared to group B (EG) was set at P <0.05 (*), P <0.01 (**), P<0.001(***)^. ^

**Table 2:** Changes in 24-hr urine creatinine, citrate, and oxalate concentrations (mg/dl) in rats (data expressed as mean±standard error).

	**Multiple Comparisons (Turkey Test)**				**ANOVA**
**Urinary parameters **	**Group A**	**Group B**	**Group C**	**Group D**	**P-value**
**Creatinine, day 0 (mg/dL) **	1.43±0.28	3.31±0.23	4.72±1.57	2.36±0.66	p>0.05
**Creatinine, day 15 (mg/dL)**	2.5±0.66	1.84±0.26	1.44±0.41	1.69±0.11	p>0.05
**Creatinine, day 30 (mg/dL)**	2.93±0.94	2.05±0.36	*0.47±0.21	1.52±0.36	p<0.05
**Citrate, day 0 (mg/dL)**	8.62±0.75	7.08±1.55	7.7±2.77	11.55±1.61	p>0.05
**Citrate, day 15 (mg/dL)**	2.87±0.08	11.16±0.33	***3.07±1.4	***4.1±1.68	p<0.001
**Citrate, day 30 (mg/dL)**	3.39±1.6	13.17±5.62	1.62±1.11	5.96±2.94	p>0.05
**Oxalate, day 0 (mg/dL)**	0.32±0.08	0.13±0.02	0.13±0.01	0.17±0.0.2	p>0.05
**Oxalate, day 15 (mg/dL)**	2.50±0.66	1.84±0.26	**0.21±0.08	**0.2±0.07	p<0.01
**Oxalate, day 30 (mg/dL)**	2.93±0.94	2.45±0.42	0.53±0.3	0.43±0.11	p>0.05

The significance level in groups C and D compared to group B (EG) was set at P <0.05 (*), P <0.01 (**), P<0.001(***)^. ^

**Table 3:** Changes in 24-hr urine volume and pH in rats (mean±standard error).

	**Multiple Comparisons (Turkey Test)**				**ANOVA**
**Urinary parameters **	**Group A**	**Group B**	**Group C**	**Group D**	**P-value**
**Urine volume, day 0 **	10±1.7	7.5±1.3	11.1±0.64	*13.8±1.74	p<0.05
**Urine volume, day 15 **	13±1.04	12±0.94	11.8±2.39	16±0	p>0.05
**Urine volume, day 30 **	14.6±1.16	12.4±1.02	*15.6±0.24	*15.6±0.24	p<0.05
**pH, day 0**	6±0	7±0	7±0	7±0	p>0.05
**pH, day 15**	6.4±0.24	6.8±0.2	6.8±0.48	5.8±0.37	p>0.05
**pH, day 30 ** **#**	6.2±0.2	*7.4±0.24	6.8±0.37	6.6±0.24	p<0.05

The significance level in groups C and D compared to group B (EG) was set at P <0.05 (*), P <0.01 (**), P<0.001(***)^.^ **# **The significance level comparison between groups A & group B (EG).

**Analysis of serum concentrations of different parameters**

**Figure 1:**
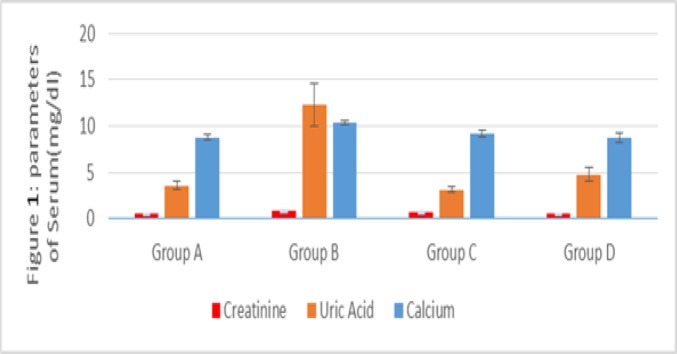
The mean serum levels of calcium, creatinine, and uric acid in groups A (control), B (1% EG), C (50 mg/kg/BW of the aqueous extract +1% EG), and D (200 mg/kg/BW of the aqueous extract +1% EG).

One-way ANOVA, followed by Tukey's test, was performed, using SPSS version 20. No significant difference was found between the studied groups in terms of creatinine concentration (p>0.05), but uric acid and calcium levels showed a significant difference. Tukey’s test reflected a significant difference in calcium level between groups A and B (p<0.05) and groups B and D (p<0.05); among all, group B had the highest calcium level. In addition, group B demonstrated a significant increase in uric acid level (p<0.01) compared to the other groups, but there was no significant intergroup difference between all the other groups (p>0.05; Figure. 1).

**Analysis of pathological status of urine tubules**

**Figure 2:**
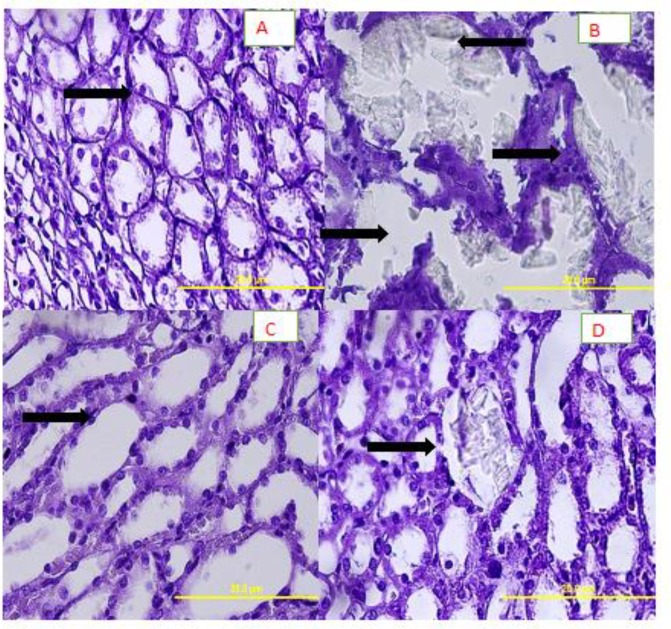
A) Group A showing normal tubules and collecting ducts (H&E×400); B) Group B showing calcium oxalate crystals and secondary renal tubular dilatation (arrows) (H&E, ×400); C) Group C (treated with 50 mg/kg of extract) presenting normal urinary tubules without calcium oxalate crystals (H&E, ×400); D) Group D (treated with 200 mg/kg of extract)showing calcium oxalate crystals in renal tubules similar to group B (H&E, ×400).

The pathological status of urinary tubules was assessed in the field view of 20.0 µm (×40 magnification). Almost no CaOx deposits or other abnormalities were found in the urinary tubules of group A (Figure 2A). On the other hand, many CaOx deposits, crystal aggregation, and various large polygonal crystals were found in different segments of the renal tubules in group B (Figure 2B). The mean number of CaOx deposits in the kidney specimens was 44±14.63 in group B as counted in 10 microscopic fields, which was significantly higher than that of group A (3±2.52) (Figures 2 A and B). 

In addition, in group C, the number of deposits was 2.2±1.42 (Figure 2C), which was significantly lower than that of groups B and A (Figures 2 A and B). The number of CaOx crystals in different parts of renal tubules was clearly higher in group D in comparison with group C. The number of oxalate deposits in group D was 42.4±18.29, which was greater in comparison with groups A and C; also, the difference was statistically significant (p<0.05; Figures A and C).

## Discussion

Based on the findings, the aqueous extract of chicory flower could reduce serum and urine calcium levels, while it resulted in an increase in urine volume. Despite the reduced level of urine calcium, the decline was only observed in the preventive group, receiving a low dose of the extract (50 mg/kg/BW; especially on day 15: p<0.01; Table 1). To the best of our knowledge, this is the first report on the effect of aqueous extract of chicory flower on the prevention of CaOx kidney stones.

Stone formation is a multi-step process, including nucleation, crystal growth, and aggregation. Calcium aggregation is a necessary step in the formation of stones. Overall, citrate seems to inhibit crystal aggregation (Gupta et al., 2011 ▶) because citric acid (citrate) circulates in the blood and forms a complex with calcium, thus, reducing the concentration of calcium oxalate at a physiological pH of 7.4. Moreover, in the present study, the significant reduction in urinary citrate (especially on day 15: p<0.001; Table 2) was the same as the decline in urine pH (on day 30: p<0.05, that is 6.6 and is less than 7.4). It is known that intracellular acidosis causes a decline in urinary citrate concentration (Gupta et al., 2011 ▶). Thus, the hypothesis, which proposes the binding of citrate to calcium, is rejected; therefore, in this study, the reduced level of urinary citrate might be associated with the reduction in pH. 

Low 24-hr urine volume can change the composition of urine and promote the risk of stone formation (Gupta et al., 2011 ▶). However, nutritional factors, such as antioxidants and vitamins, can be effective in reducing stone formation. In this study, the effect of aqueous extract of chicory flower on calcium oxalate stone formation in male Wistar rats (group C : low-dose group) receiving EG, might be due to the presence of antioxidant compounds, such as steroids, terpenoids, vitamins, anthocyanins, flavonoids, polyphenols, alkaloids, and tannins in the flower extract (Al-Snafi et al., 2016 ▶). In urinary tubules, the stone count and accumulation of calcium crystals reduced in the preventive group receiving 50 mg/kg of the extract. 

Therefore, considering that the 200 mg/kg dose of the extract did not significantly decrease the urinalysis parameters, especially calcium and creatinine, it seems that high doses of the extract can have a negative effect on formation of calcium oxalate. Moreover, chicory flower extract was more effective in reducing the formation of calcium oxalate urinary stones compared to uric acid stones, as it did not significantly decrease the urinary level of uric acid.

 Overall, calcium oxalate crystals can damage the surface cells of kidney tubes. Damage to these cells, caused by free radicals, can also induce heterogeneous nucleation of crystals (Khan and Thamilselvan, 2011 ▶). Flavonoids and phenolic acids, due to the presence of hydroxyl groups in their structure and their contribution to defence mechanisms against oxidative damage, are extremely important (Shad et al., 2013 ▶) as they can provide marked protection against oxidative and free radical damage (Rashedet al., 2016 ▶). 

Although the exact mechanism involved in increased urine volume and reduced serum and urinary levels of calcium (50 mg/kg/BW) has not been determined, the high volume of urine seems to reduce the relative supersaturation of crystal-forming components. It also indicates a high urine flow rate, which tends to wash out any formed crystals (Gupta et al., 2011 ▶; Zaman and Noorul Basar, 2013 ▶).

Chicory flower (50 mg/kg/BW) may play a preventive role against renal stone formation due to its diuretic, antibacterial, and anti-inflammatory effects (Shad et al., 2013 ▶). Also, increased levels of parameters contributing to the prevention of kidney stone formation, such as nephrocalcin (which is produced in the human kidney and inhibits calcium oxalate crystal growth), urinary prothrombin fragment 1 (UPTF1) (Gupta et al., 2011 ▶), THP, or uromodulin (linked to water/electrolyte balance), may be involved in the protective effects of this plant against urinary tract infections (Rampoldi et al., 2011 ▶). It should be noted that increased concentration of THP inhibits nucleation, aggregation of CaOx stones, and crystal adhesion to renal cells (Hoyer et al., 1979). The mentioned mechanisms and the presence of antioxidants in chicory may prevent the release of various inflammatory factors, which contribute to heterogeneous nucleation and inhibit the binding of calcium oxalate crystals and stone formation (El-Dakhakhny et al., 2002 ▶).

Based on the present findings, chicory flower extract might play a role in the prevention of kidney stone formation as it decreased the urinary level of oxalate while increased the urinary levels of calcium and creatinine at a high dose (200 mg/kg/BW). Although low doses of chicory flower extract might affect the formation of calcium stones, determining the exact effect of chicory flower and the mechanisms involved in stone formation require further research.
